# Ferroptosis-related *lncRNA NRAV* affects the prognosis of hepatocellular carcinoma via the *miR-375-3P/SLC7A11* axis

**DOI:** 10.1186/s12885-024-12265-y

**Published:** 2024-04-18

**Authors:** Ke Zong, Caifeng Lin, Kai Luo, Yilei Deng, Hongfei Wang, Jianfei Hu, Shi Chen, Renfeng Li

**Affiliations:** 1 Department of Hepatobiliary Surgery, The First Affiliated Hospital of Zhengzhou University, Zhengzhou, Henan 450052 China; 2 Shengli Clinical Medical College of Fujian Medical University, Fujian Medical University, No. 134, East Street, Fuzhou, Fujian Province 350001 PR China; 3 Department of Hepatopancreatobiliary Surgery, Fujian Provincial Hospital, No. 134, East Street, Fuzhou, Fujian Province 350001 PR China

**Keywords:** lncRNA, Ferroptosis, Predictive models, *NRAV*, *miR-375-3P*/*SLC7A11*

## Abstract

Ferroptosis has important value in cancer treatment. It is significant to explore the new ferroptosis-related lncRNAs prediction model in Hepatocellular carcinoma (HCC) and the potential molecular mechanism of ferroptosis-related lncRNAs. We constructed a prognostic multi-lncRNA signature based on ferroptosis-related differentially expressed lncRNAs in HCC. qRT-PCR was applied to determine the expression of lncRNA in HCC cells. The biological roles of *NRAV* in vitro and in vivo were determined by performing a series of functional experiments. Furthermore, dual-luciferase reporter and RNA immunoprecipitation (RIP) assays were used to confirm the interaction of *NRAV* with *miR-375-3P*. We identified 6 differently expressed lncRNAs associated with the prognosis of HCC. Kaplan–Meier analyses revealed the high-risk lncRNAs signature associated with poor prognosis of HCC. Moreover, the AUC of the lncRNAs signature showed utility in predicting HCC prognosis. Further functional experiments show that the high expression of *NRAV* can strengthen the viciousness of HCC. Interestingly, we found that *NRAV* can enhance iron export and ferroptosis resistance. Further study showed that *NRAV* competitively binds to *miR-375-3P* and attenuates the inhibitory effect of *miR-375-3P* on *SLC7A11*, affecting the prognosis of patients with HCC. In conclusion, We developed a novel ferroptosis-related lncRNAs prognostic model with important predictive value for the prognosis of HCC. *NRAV* is important in ferroptosis induction through the *miR-375-3P*/*SLC7A11* axis.

**Supplementary Information:**

The online version contains supplementary material available at 10.1186/s12885-024-12265-y.

## Introduction

Hepatocellular carcinoma (HCC) is a common malignant tumor of the digestive system worldwide. The incidence of HCC is on the rise worldwide, causing approximately 830,000 deaths each year, ranking third among the causes of cancer death [1]. Treatments such as hepatectomy, liver transplantation, microwave ablation, and systemic therapy have advanced. However, due to the limited means of early detection, HCC's diagnosis and curative effect are still unsatisfactory, and the 5-year survival rate is only 18% [2]. It is exciting that the success of immunotherapy in recent years has ushered in a new era of cancer treatment. Immunotherapy has also achieved a remarkable curative effect on HCC, including the application of PD-1 monoclonal antibodies and CTLA-4 monoclonal antibodies. Therefore, continuing research on immunity has positive implications for patients.

Regulatory cell death (RCD) is a mode of cell death controlled by specific signal transduction pathways. The most widely studied RCD types include apoptosis, pyroptosis, necrosis, autophagy, and ferroptosis, each of which has its unique molecular mechanism. Ferroptosis is a new type of iron-dependent regulatory cell death. Regarding mechanism, ferroptosis is an iron-dependent high expression of unsaturated fatty acids on the cell membrane, resulting in lipid peroxidation and induced cell death. Exogenous or endogenous pathways can induce Ferroptosis. The exogenous pathway is initiated by inhibiting cell membrane transporters, and the endogenous pathway is initiated by blocking the activation of intracellular antioxidant enzymes [3]. The effect of ferroptosis on the efficacy of chemotherapy, radiotherapy, and immunotherapy has been determined [3, 4]. In the process of tumorigenesis, ferroptosis plays a dual role in promoting and inhibiting tumors, which depends on the release of damage-associated molecular patterns (DAMPs) can mediate immunogenic cell death that could stimulate antitumor immunity, so the combined use of drugs for ferroptosis signals can improve the effectiveness of these therapies [5]. It is an indisputable fact that ferroptosis can enhance the therapeutic effect of immunotherapy [6]. However, many underlying molecular mechanisms remain unclear. Therefore, it is still necessary to strengthen the research on ferroptosis. Strengthening the study of ferroptosis is necessary to improve clinical prognosis.

LncRNA is defined as a noncoding RNA of more than 200 bp. Current studies have shown that dysfunctional lncRNA plays an important role in tumorigenesis through many cancer-related biological processes, including apoptosis [7], cell cycle, metastasis, and DNA damage response [8, 9]. In recent years, increasing evidence has shown that lncRNAs are closely related to ferroptosis. The lncRNA LINC00336 inhibits ferroptosis in lung cancer by acting as a ceRNA by Wang et al. [10]. LINC00336 overexpression significantly reduced intracellular Fe^2+^ and lipid ROS. Mao et al. found that the p53-related lncRNA P53RRA can directly interact with the functional domain of signaling proteins in the cytoplasm by activating the p53 pathway, promoting ferroptosis, and playing a tumor suppressor role [11]. Therefore, the study of lncRNA related to ferroptosis and HCC is very important for understanding the mechanism of tumor development.

Our study first constructed a prognostic multi-lncRNA signature of differentially expressed ferroptosis-related lncRNAs based on the Cancer Genome Atlas (TCGA) data and International Cancer Genome Consortium (ICGC) data, further verifying the model's potential role in immunotherapy to combine ferroptosis and immunotherapy and increasing the possibility of clinical transformation. Furthermore, in combination with clinicopathological features, a nomogram based on our gene signature presented improved predictive power and risk stratification for HCC. Then we verified the key genes in the prognosis model and the role of *NRAV* in the process of ferroptosis in vitro and in vivo. Our findings establish a lncRNA prognostic model related to ferroptosis based on the expression of lncRNA, which complements the mechanism of lncRNA regulating ferroptosis in HCC.

## Method

### Data collection

The RNA sequences of 422 patients (50 normal tissues and 374 tumor tissues) were extracted from the TCGA-HCC database, and the RNA sequences of 445 patients (202 normal tissues and 243 tumor tissues) were extracted from the ICGC-LIRI-JP database. Ferroptosis-related genes were acquired from FerrDb, an online source that supplies a comprehensive and updated checklist of ferroptosis markers, regulative molecules, and ferroptosis-disease associations [12]. In general, we identified 175 (Driver:76; suppressor:47; marker:75) ferroptosis-related genes (Table S1). Pearson correlation evaluation was used to identify the relationships between ferroptosis-related genes and lncRNAs. Connections with the value of correlation coefficients |R|> 0.5 and a *P*-value < 0.001 were considered statistically substantial. The clinicopathological attributes of HCC people, consisting of age, gender, stage, TMN, survival condition, and survival time, were accumulated. In this study, we identified significant differentially expressed lncRNAs (DElncRNAs) between HCC tumors and normal liver tissue (absolute log2FC >  = 1 and FDR < 0.05). After screening differentially expressed genetics (DEGs), we performed functional enrichment evaluation and KEGG analysis using upregulated and downregulated ferroptosis-related differentially expressed genetics (DEGs). Enrichment analysis using metascape for EDGs data.

### Building of the ferroptosis-related lncRNAs prognostic signature

Univariate Cox regression, LASSO, and multivariate Cox regression evaluations were used to establish the last variables for building the prognostic threat score model. stratified based on risk rating (Coefficient lncRNA1 × expression of lncRNA1) + (Coefficient lncRNA2 × expression of lncRNA2) + ⋯ + (Coefficient lncRNAn × expression lncRNAn). Based on the threat scores for OS, HCC clients with fibrosis were separated into high- or low-risk groups using the typical score as a cut-off, and also Kaplan–Meier contours were calculated for both teams.

### Predictive nomogram building and validation

To execute genetics established enrichment evaluations stabilized, all genetics matters were imported right into the GSEA software application as well as the Molecular Signature Database (MSigDB) Trademark, KEGG, and also Genetics Ontology gene libraries were utilized to identify enriched gene sets [13], which were then searched in the TCGA-HCC database. Enriched gene sets were selected based on statistical significance (incorrect exploration rate FDR q value < 0.25 and normalized *p*-value < 0.05) [14]. We built a nomogram incorporating typical medical variables such as age, gender, grade, tumor stage, and the risk rating originated from the prognostic trademark to assess the possible 3- and 5-year OS of clients with HCC.

### Gene expression and immunity analysis

At the same time, the CIBERSORT [15, 16], ESTIMATE [17], quanTIseq [18], xCell [19], MCP-counter (or mMCP-counter for mouse) [20], EPIC [21], and TIMER [22] formulas were analyzed, the CC or cell sorts of immune reactions in heterogeneous examples among risky as well as low-risk teams based on ferroptosis-related lncRNAs trademark. The differences in immune feedback under various formulas were revealed using a Heatmap. In enhancement, ssGSEA was utilized to quantify the tumor-infiltrating immune cell subgroups between both groups and assess their immune function. Many possible immune check factor molecules were determined that could be targeted and incorporated with potential medication therapy [23].

### Cell culture and transfection

Liver cancer cell lines (HuH-7, SKhep1, hep3B, hepG2) were ordered from the Chinese Academy of Sciences (Shanghai, China) and cultured in DMEM medium containing 25 mM glucose, 4 mM L-glutamine and 1 mM pyruvate (Gibco, 12800). All cell lines were authenticated using short tandem repeat (STR) profiling (by GENEWIZ Co. Ltd. at Suzhou, China). After authentication, large frozen stocks were made for future use. All cell lines were used within 15 passages (less than 2 months)after reviving from the frozen stocks. The medium was supplemented with 10% FBS and 1% penicillin/streptomycin. Cells were kept at 37 °C in a humidified incubator with 5% CO_2_. The trypan blue exclusion assay was used to detect cell growth. All cell lines were tested for mycoplasma contamination, and no cell lines were contaminated. The *miR-375-3P* mimic, inhibitor, and corresponding control oligonucleotides were purchased from Shangya (Fuzhou, China). The transfection of *miR-375-3P* mimics, inhibitors, or corresponding NCs into cells was performed using Lipofectamine 3000 (Invitrogen). The lentiviruses of Flag-SRSF5, NRAV, overexpression vector (vector), sh-NRAV, and empty vector (sh-NC) were constructed by Genechem (Shanghai, China). The cells were transfected with lentivirus according to the manufacturer's instructions. The sequences of shRNA against specific targets are available in Table S2.

### Nucleocytoplasmic separation

Total RNAs were extracted from cell lines and tissues using Trizol (Invitrogen, USA). The RNA of nuclear and cytoplasmic was separated and extracted using PARIS Kit (Life technologies, USA) according to the manufacturer’s instructions. The expression levels of NRAV, U6, and GAPDH genes in various RNA samples obtained by qRT-PCR were measured.

### Tissue specimens

Eighty-six matched HCC tissues and paracancerous tissues were obtained from patients with HCC diagnosed by The First Affiliated Hospital of Zhengzhou University. All experiments involving human samples and clinical data were approved by the Accreditation Committee of The First Affiliated Hospital of Zhengzhou University.

### RNA extraction and quantitative real-time PCR (qRT-PCR) analysis

According to the manufacturer's protocol, total RNA from HCC cells, tissues, and matched non-cancerous tissues was isolated using the TRIzol Reagent (Thermo Fisher Scientific, Waltham, MA, USA). Reverse transcription was performed using the PrimeScript RT Reagent Kit (Takara, Dalian, China). Bulge-loop™ miRNA RT-qPCR Primers were applied to determine the level of miRNAs. The real-time PCR reactions were performed using StepOnePlus™ Real-Time PCR System (Thermo Fisher Scientific, MA, US). The program settings on temperature cycling were followed as instructed by the manufacturer. GAPDH was the cytoplasmic control, and U6 was the nuclear control. The sequences of primers are listed in Table S2.

### Western blot analysis

In brief, proteins were isolated from HCC cells using RIPA buffer (Solarbio, Beijing, China) supplemented with proteinase inhibitors, and the protein concentration was determined with a BCA reagent (Beyotime, Beijing, China). Cell lysates were separated on SDS–polyacrylamide gels and then transferred onto polyvinylidene difluoride (PVDF) membranes (Millipore). After the membranes were blocked in 5% skim powdered milk for 2 h, they were incubated with primary antibodies overnight at 4 °C. The primary antibodies used in this study included: ACSL4 (HUABIO, ET7111-43), GPX4 (HUABIO, ET1706-45), *SLC7A11* (Abcam, ab175186), and GAPDH (Proteintech, 80,570–1-RR), FLAG(Sigma-Aldrich, No. F7425). Next, the membranes were incubated with secondary antibodies (HUABIO, Hangzhou, China) at room temperature for 1 h. After washing three times, the targeted proteins were visualized using enhanced chemiluminescence (ECL) reagent (Millipore, MA, USA). GAPDH was used as the loading control in this study. Flag-SRSF5 served as a positive control.

### Colony formation assay

Cells were seeded into 12-well plates at an initial density of 700 cells/well and cultured for 2 weeks. The colonies were fixed in 4% paraformaldehyde and stained with 0.1% crystal violet (Sigma, St. Louis, MO, USA). The visible colonies were counted using a light microscope. The number of colonies in triplicate wells were measured for each treatment group.

### Transwell migration assays

Transwell assays were conducted using 24-well plates with chamber inserts with 8.0 μm pores (Corning). The cells were digested 24 h after transfection and resuspended in a serum-free medium. Then, 3 × 10^4^ cells per well were placed into the upper chambers. μlLike a cell nutritional attractant, the lower chamber was filled with a 500 μl medium with 10% FBS. After incubation at 37 °C for 24 h, cells in the upper chamber were gently removed with a cotton swab. The migrated or invaded cells were fixed with 4% polyformaldehyde and visualized by staining with crystal violet for 20 min. Last, the migrated cells were imaged and quantified by capturing five randomly chosen microscopic fields using an inverted microscope (Olympus, Japan). All experiments were performed in triplicate.

### Cell proliferation assays

For the Cell Counting Kit-8 (CCK-8) assay, the treated HCC cells were seeded into 96-well plates at a concentration of 3 × 10^3^ /well. Then, 10 μl of CCK-8 assay solution (Dojindo, Japan) was added and incubated in the dark for 2 h. The absorbance at 450 nm was measured every 24 h with a microplate reader (BioTek Instruments, USA). All experiments were repeated three times.

### RNA immunoprecipitation (RIP)

Following the manufacturer's protocol, RNA immunoprecipitation (RIP) assay was performed using Magna RIP™ RNA-binding protein immunoprecipitation kit (Millipore, Billerica, MA, USA). The cell extract was incubated with magnetic beads conjugated with anti-Argonaute 2 (AGO2) or anti-IgG antibody (Millipore, Billerica, MA, USA) for 6 h at 4 °C. The beads were washed and incubated with Proteinase K to remove proteins. Finally, isolated RNA was extracted using TRIzol Reagent (Thermo Fisher Scientific, Waltham, MA, USA), then the purified RNA was subjected to qRT-PCR analysis. The specific binding sites of NRAV and *miR-375-3P* were determined by annolnc2 (http://annolnc.gao-lab.org).

### Dual-luciferase reporter assay

The full-length wild-type (WT) sequence of *NRAV* and the indicated mutant *NRAV* containing the predicted *miR-375-3P* binding sites were separately synthesized and cloned into the dual-luciferase reporter vector (Genechem, Shanghai, China). The resulting dual-luciferase reporter plasmids (WT or Mut) were co-transfected with the *miR-375-3P* mimic or inhibitor into hepG2 or HuH-7 cells, respectively, using Lipofectamine 3000. After 48 h of incubation, the relative firefly luciferase activities concerning the corresponding Renilla luciferase activities were measured and analyzed using a Dual-Luciferase Assay System (Promega, Fitchburg, WI, USA) following the manufacturer's protocol.

### Iron assay

Intracellular ferrous iron (Fe^2+^) level was determined using the iron assay kit (ab83366, Abcam) according to the manufacturer's instructions. Cells were collected and washed in ice-cold PBS, homogenized in 5 × volumes of iron assay buffer on ice, then centrifuged (13,000 × g, 10 min) at 4 °C to remove insoluble material. The supernatant was collected, and an iron reducer was added to each sample before mixing and incubating at room temperature for 30 min. Then, 100 μl of the iron probe was added to each sample, mixing and incubating the reaction for 1 h at room temperature in the dark. The absorbance at 593 nm was measured immediately using a colorimetric microplate reader.

### Lipid ROS measurement

Lipid ROS level was analyzed by flow cytometry using BODIPY-C11 (GLPBIO, GC40165) dye. Cells were seeded at 2.5 × 10^5^ per well in a six-well dish and grown overnight for 12 h. Cells were washed once with PBS. Cells were then stained with 2 ml medium containing 5 µM of BODIPY-C11 and incubated at 37 °C for 20 min in the dark. Cells were washed twice with PBS to remove excess labeling mixture, followed by resuspending in 500 μl fresh PBS (DPBS, Gibco). The cell suspension was filtered through a 0.4 μM cell filter and subjected to flow cytometric analysis to detect the amount of intracellular lipid ROS. The fluorescence intensities of cells per sample were determined by flow cytometry using the BD FACSAria cytometer (BD Biosciences). A minimum of 10,000 cells was analyzed for each sample. Data analysis was evaluated using the FlowJo Software.

### Subcutaneous xenograft model

For the subcutaneous xenograft mouse model, 10 4- to 6-week-old male athymic BALB/c nude mice (Shanghai SLAC Laboratory Animal Co,Ltd., China) were housed and fed in standard pathogen-free conditions. The male nude mice were randomly divided into two groups (*n* = 5 in each group) and inoculated with cells as follows: sh-NC stable transfected hepG2 Cell (2 × 10^6^ cells); sh-*NRAV* stable transfected hepG2 Cell (2 × 10^6^ cells); Cells were mixed with matrigel (1:2) and inoculated subcutaneously at the right rear back region. Tumors were measured weekly with calipers, and tumor volume was calculated as = (L × W^2^)/2. Five weeks later, the mice were euthanized and the tumors were isolated for further study. If a tumor grew to 2000 mm^3^, the experiment was terminated in advance. After measurement, the mice were euthanized with an overdose of 10% pentobarbital sodium (100 mg/kg; intraperitoneal injection), and death was confirmed by the disappearance of the heartbeat. All in vivo animal experiments were approved by the Committee on the Ethics of Animal Experiments at the Shanghai University of Traditional Chinese Medicine.

### Statistical analysis

The experimental data were analyzed using statistical analysis software, including GraphPad Prism 8.0 software (GraphPad) and the R package (V.3.3.4). Differences between the indicated groups were compared using the Student's *t*-test and one-way analysis of variance (ANOVA) followed by Fisher's least significant difference (LSD) test. Correlations were evaluated by Pearson correlation analysis. The cumulative overall survival (OS) and progression-free survival (PFS) rates were calculated using the Kaplan–Meier method, and significance was evaluated with the log-rank test. A *P*-value < 0.05 was considered to indicate a statistically significant result.

## Result

### Identification of robust DEGs

**Fig. 1:**
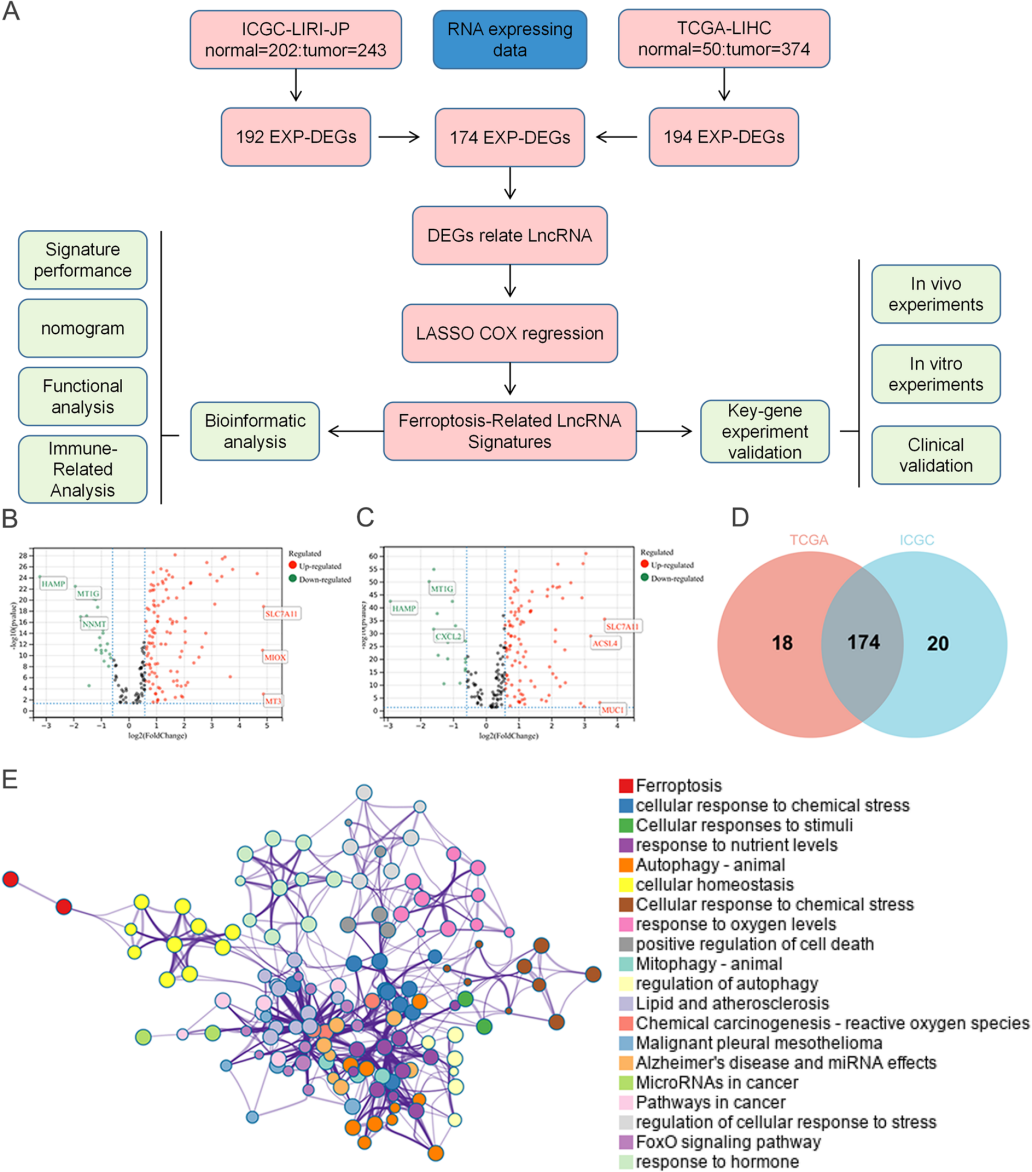
**A** The study flowchart. **B**-**E** A screen of the ferroptosis-related differentially expressed genes in HCC

The ferroptosis-related lncRNAs signature was constructed following the flowchart shown in Fig. 1A. We uncovered 174 ferroptosis-related DEGs (38 downregulated and 136 upregulated; Fig. 1B-D, Table S1). Enrichment analysis revealed the over-expressed genes were mainly involved in ferroptosis, cellular response to chemical, oxidative, and oxidative stress. (Fig. 1E).

### Construction and validation of ferroptosis-related lncRNA prognostic model

**Fig. 2:**
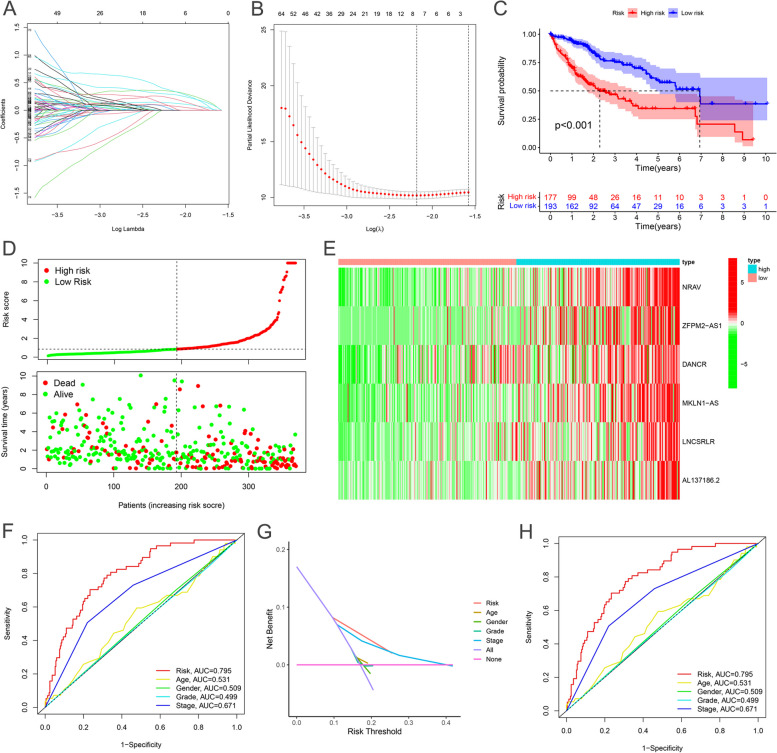
Ferroptosis-related lncRNAs signature for prognostic prediction. **A**, **B** LASSO regression model of the prognostic ferroptosis-related lncRNAs. **C** Kaplan–Meier curves resulted from the log-rank test for survival Analyses. **D**, **E** Risk survival status plot. **F** The AUC values of the risk models. **G** The DCA of the risk models. **H** The AUC predicts HCC patients' 1, 3, and 5-year survival rates

A prognostic risk evaluation model based on only 6 ferroptosis-related lncRNAs was then constructed using the optimal penalty parameter (λ = -1.8) for the LASSO model from the ferroptosis-related lncRNAs group. The cvfit and lambda curve are shown in Fig. 2A, B. Kaplan–Meier curve analysis also demonstrated that patients with the high-risk lncRNA signature had much poorer survival than those with the low-risk lncRNA signature (*P* < 0.001, Fig. 2C). Figure 2D demonstrated the variation of risk scores between the low-risk and high-risk groups and more fatalities and fewer years of survival in the high-risk group. As shown in Fig. 2E, the RNA expression of the six ferroptosis-associated lncRNAs was lower in the low-risk group than in the high-risk group. Meanwhile, the AUC of the signature lncRNAs was 0.795. Then, we demonstrated that the 6-gene signature was an independent prognostic factor for HCC patients superior to traditional clinicopathological factors and verified their survival prediction ability in an external HCC cohort in TCGA (Fig. 2F, G). The AUC predictive value of the novel lncRNAs signature for 1, 3, and 5-year survival rates was 0.795, 0.749, and 0.748, respectively (Fig. 2H).

**Fig. 3:**
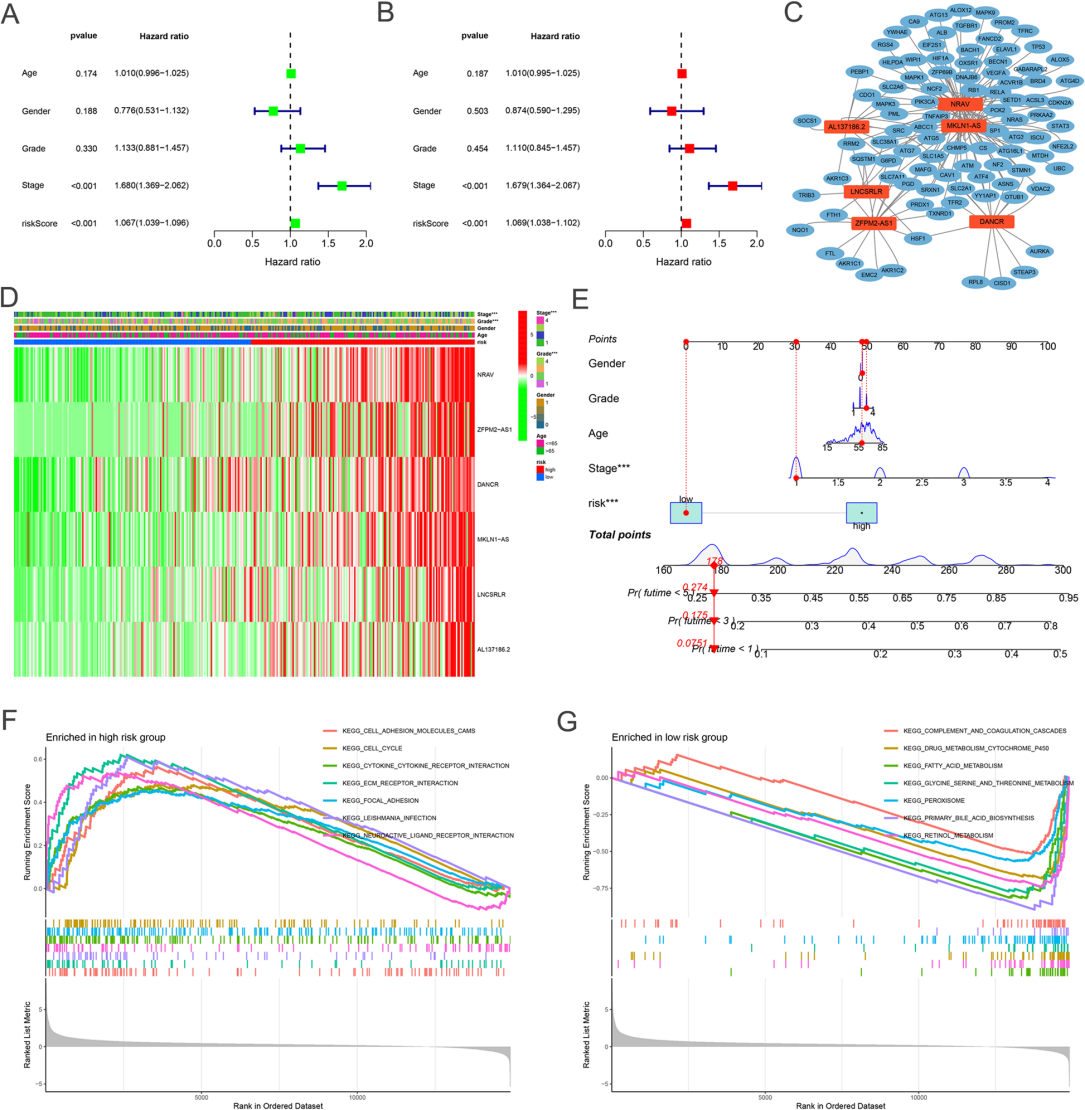
The independent prognostic value. **A**, **B** The forest plots for univariate and multivariate Cox regression analysis. **C** The relationship between the novel lncRNAs and Ferroptosis-related genes. Heatmap for ferroptosis-related lncRNAs prognostic signature and clinicopathological manifestations. **D** Heatmap for ferroptosis-related lncRNAs prognostic signature and clinicopathological manifestations. **E** A nomogram model combines both clinical factors and prognostic ferroptosis-related lncRNAs. **F**, **G** Gene enrichment analysis

Subsequently, we used univariate Cox analysis, a Robust likelihood-based survival model, and multivariate Cox analysis to construct a signature composed of lncRNAs and tumor stage, independent prognosis factors of OS of HCC patients (*P* < 0.001; Fig. 3A, B). The relationship between the novel lncRNAs and Ferroptosis-related genes is shown in Fig. 3C. The heatmap for the association between ferroptosis-related lncRNAs prognostic signature and clinicopathological manifestations was also analyzed (Fig. 3D). The hybrid nomogram incorporating clinicopathological characteristics and the novel ferroptosis-related lncRNAs prognostic signature (Fig. 3E) was stable and accurate, thus may be applied in surgical pathology or clinical management of HCC patients. Gene set enrichment analyses (GSEA) revealed that most of the novel ferroptosis-related lncRNAs of high-risk groups' prognostic signature regulated cell cycle and tumor-related pathways (Fig. 3F). On the other hand, most of the novel ferroptosis-related lncRNAs of low-risk groups' prognostic signatures regulated multiple metabolic processes of cells (Fig. 3G). The heatmap of immune responses based on CIBERSORT, ESTIMATE, quanTIseq, xCell, MCP-counter (or mMCP-counter for mouse), EPIC, and TIMER algorithms is shown in Figure S1A. To further explore the relationships between the risk scores and immune cells and functions, we quantified the enrichment scores of 16 immune cell subpopulations and their related functions with the ssGSEA R package, which revealed that APC co-stimulation, cytolytic activity, MHC class I, type I INF response and type II INF response were significantly different between the low-risk and high-risk groups (Fig. S1B). Given the importance of checkpoint inhibitor-based immunotherapies, we further explored the difference in the expression of immune checkpoints between the two groups. We found a significant difference in the expression of HHLA2, TNFRSF14, and CTLA4 among other groups of patients (Fig. S1C).

### Identification of *NRAV* in HCC

**Fig. 4:**
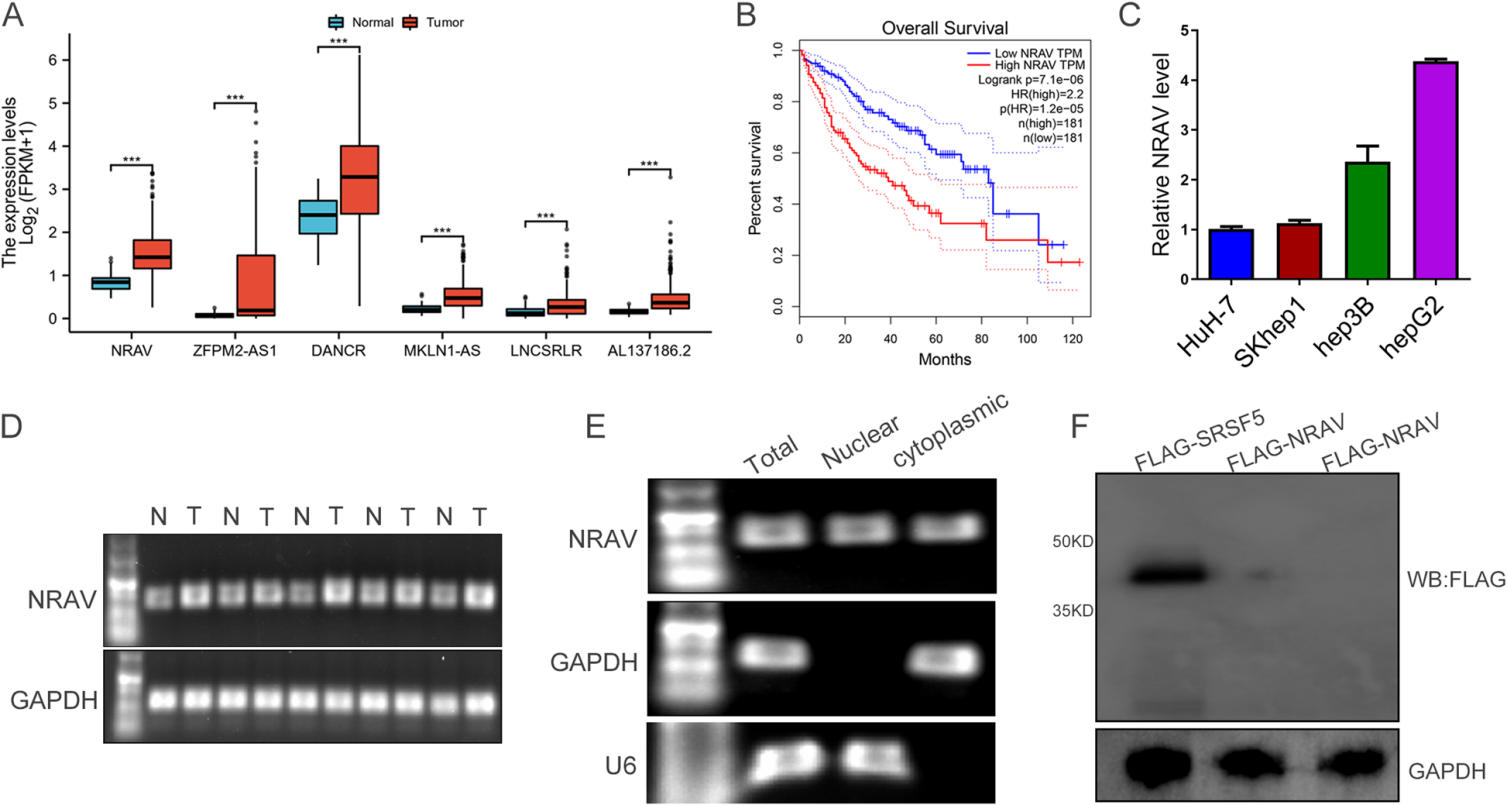
Identifying *NRAV*. **A** Expression levels of 6 selected genes between tumors and peritumor tissues in TCGA database. **B** Overall survival of HCC patients from gepia with high or low *NRAV* expression levels. **C** The expression levels of *NRAV* in HCC cells (HuH-7, SKhep1, hep3B, hepG2) **D**. Quantitative real-time PCR was performed to examine the expression of *NRAV* in tumors and matched non-tumor tissues from HCC patients. **E** RT-PCR was performed to examine cytoplasmic or nuclear *NRAV* RNA levels in hepG2 cells. GAPDH was the cytoplasmic control, and U6 was the nuclear control. **F** The constructs were transfected into HEK293T cells for 48 h with an N-terminal Flag tag. Cell lysates were harvested and subjected to Western blotting with Flag antibody. Flag-SRSF5 served as a positive control. Results are presented as mean ± SD. *P < 0.05; **P < 0.01, ***P < 0.001, ****P < 0.0001

Next, to further confirm the role of lncRNAs in the HCC prognostic model. By analyzing the data of 424 cases of HCC in the TCGA database, the expression levels of 6 ferroptosis-related lncRNAs are shown in Fig. 4A. Through the prognostic analysis of three molecules in the Gepia database, we found that *NRAV* was significant for OS (Fig. 4B), while *DANCR*, *MKLN1-AS1*, and *ZFPM2-AS1* showed little difference (Fig. S2A-C), and no difference between *AL137186.2* and *LNCSRLR*. So we selected *NRAV*s with the most significant difference in HCC prognosis for further validation. *NRAV* is located on chromosome 12. In different HCC cell lines, the expression of *NRAV* is shown in Fig. 4C. Compared with Paracancer, *NRAV* is generally upregulated in cancer and can be stably expressed in various commercial HCC cells (Fig. 4D). The subcellular localization of *NRAV* in hepatoma cells was detected by nuclear and cytoplasmic separation experiments, and it was found that *NRAV* was located not only in the cytoplasm but also in the nucleus (Fig. 4E). With the deepening of the research, researchers have found that many noncoding RNAs can also work by translating into proteins. To study the action mode of *NRAV*, we use software to predict and analyze the protein-coding ability of *NRAV*. ORF reading frame prediction shows a reading frame, and ORF12 is greater than 300 bp (Fig. S2D), arousing our strong curiosity. However, it is a pity that coding-potential computer (CPC) analysis shows that the coding possibility of *NRAV* is very little (Fig. S2E), and we can not detect any special protein bands through in vitro translation experiments, while the control gene can detect band (Fig. 4F), which also supports that *NRAV* is a noncoding RNA.

### *NRAV* promotes HCC tumorigenesis in vitro and in vivo

**Fig. 5:**
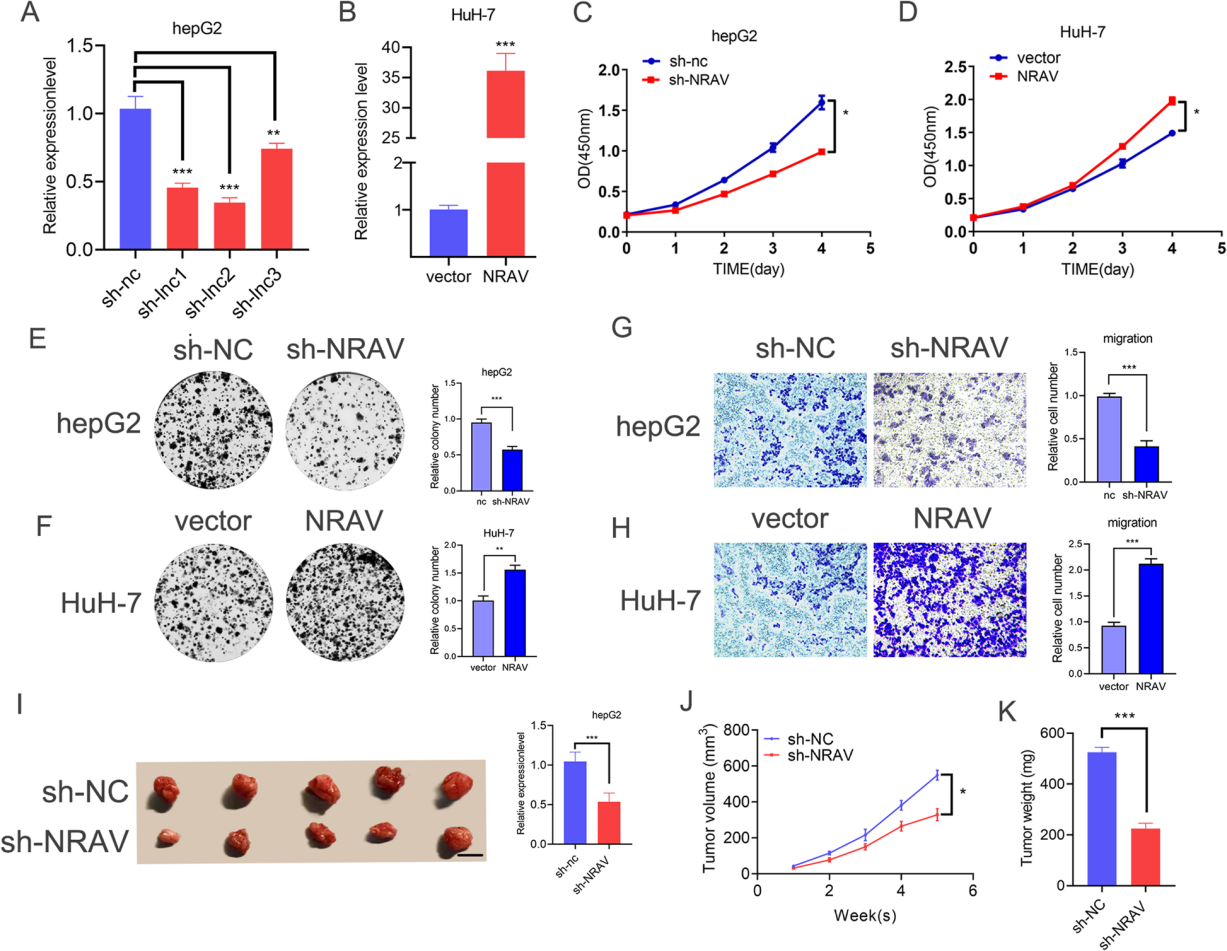
*NRAV* functions as an oncogene in HCC. **A** The expression of *NRAV* in hepG2 cells was analyzed by qRT-PCR after transfection with three siRNAs or the control siRNA (si-NC). **B** The levels of *NRAV* in HuH-7 cells were analyzed by qRT-PCR after stable transfection with the *NRAV* overexpression or the control vector. **C**-**F** Cell proliferation was assessed by CCK-8 (**C**, **D**) and colony formation (**E**, **F**). **G**-**H** Transwell assays confirmed that the migration ability of the HCC cell line was suppressed with *NRAV* knockdown (**G**) and enhanced with *NRAV* overexpression (**H**). **I**-**K** Representative images of tumors from nude mice inoculated with HepG2 cells (NRAV knockdown or control group) and *NRAV* knockdown decreased tumor volume and tumor weight of nude mice. Scale bars, 1 cm. Results are presented as mean ± SD. *P < 0.05; **P < 0.01, ***P < 0.001, ****P < 0.0001

Next, we studied the carcinogenic function of *NRAV* in HCC. To evaluate the biological function of *NRAV* in HCC cells, we designed and constructed three siRNA targeting *NRAV* to change their expression specifically. QRT-PCR results showed that all three siRNAs effectively silenced *NRAV* (Fig. 5A). The introduction of the recombinant human *NRAV* gene successfully realized the overexpression of the gene (Fig. 5B). The results of CCK-8 and clone formation experiments showed that knockout of the *NRAV* gene effectively inhibited the proliferation of hepG2, while up-regulation of *NRAV* significantly enhanced the proliferation of HuH-7 (Fig. 5C-F). Transwell's experiment showed that the down-regulation of *NRAV* significantly decreased the migration ability of hepG2, while the up-regulation of *NRAV* enhanced the migration ability of HuH-7 cells (Fig. 5G, H). Next, we constructed a CDX model to examine the carcinogenic function of *NRAV *in vivo. Compared with the control group, the tumor volume and weight in the knockout group decreased significantly, and the tumor growth rate slowed (Fig. 5I-K). To sum up, these experimental results show that *NRAV* has a carcinogenic function in HCC.

### *NRAV* promotes iron export and ferroptosis resistance as a sponge of *miR-375-3P*

**Fig. 6:**
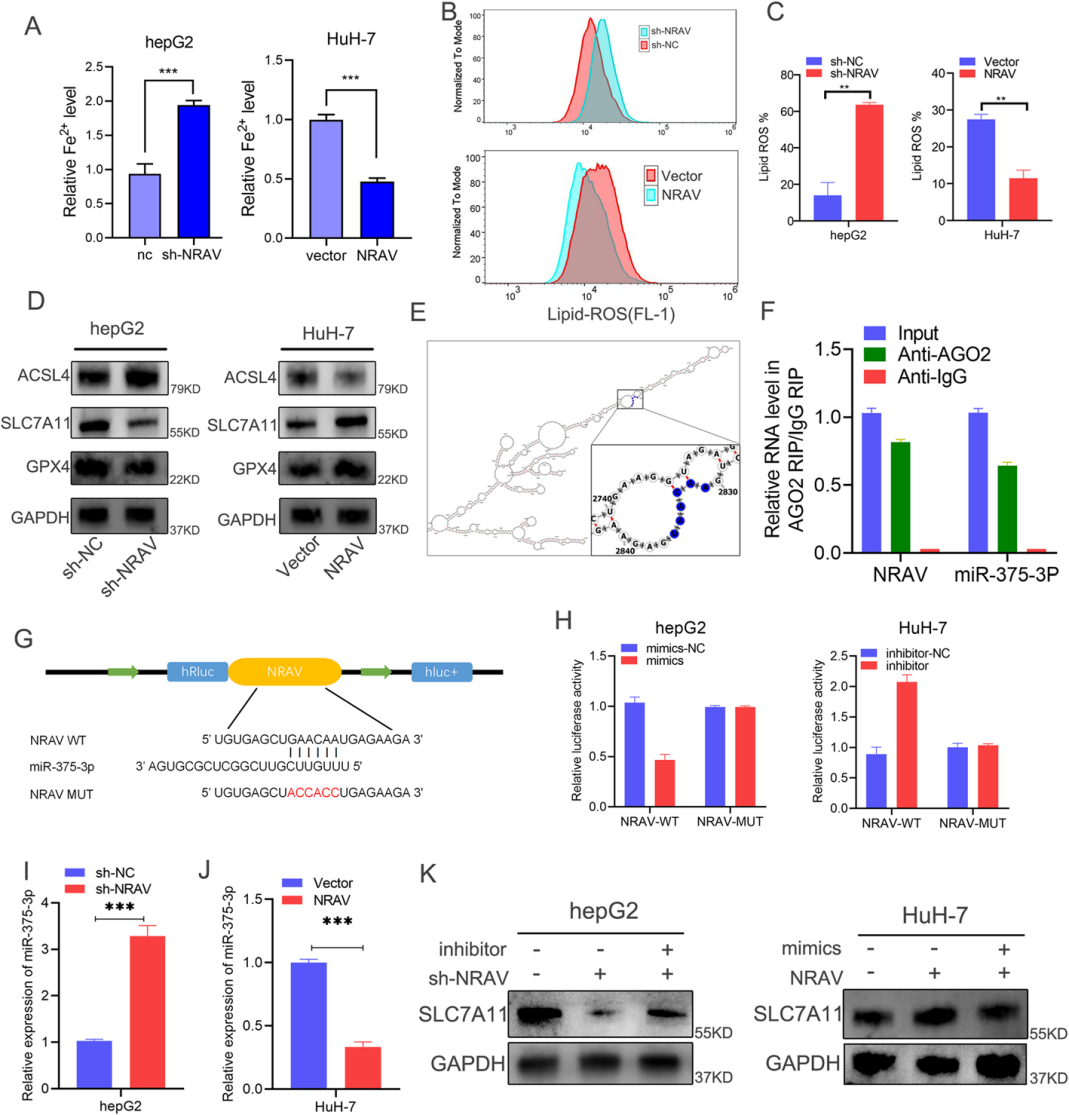
*NRAV* induces iron export and ferroptosis resistance via sponging *miR-375-3P* in HCC. **A** Iron content increased after *NRAV* knockdown and decreased after *NRAV* overexpression. **B**-**C** Flow cytometry of ROS levels in HCC cells after *NRAV* knockdown or overexpression. **D** Western blot showed expressions of the ferroptosis-related proteins (ACSL4, SCL7A11, and GPX4) in hepG2 Cell with *NRAV* knockdown and HuH-7 Cell with *NRAV* overexpression and control. **E** *NRAV* and *miR-375-3P* binding to the secondary structural diagram from AnnoLnc. **F** RIP assay confirmed the interaction of *miR-375-3P* with *NRAV* in HCC cells. **G** Schematic illustration of the *NRAV*-WT and *NRAV*-Mut luciferase vectors. **H** Relative luciferase activities in hepG2 and HuH-7 cells co-transfected with *NRAV*-WT or *NRAV*-Mut and the *miR-375-3P* mimic, inhibitor, or corresponding negative control. **I**-**J** The expression level of *miR-375-3P* in each group transfected was verified through RT-qPCR **K**. The *SLC7A11* protein levels in hepG2 and HuH-7 cells from different groups were determined by western blot analysis. GAPDH was used as a control. Results are presented as mean ± SD. *P < 0.05; **P < 0.01, ***P < 0.001, ****P < 0.0001

Iron accumulation was increased in hepG2 cells infected with sh-*NRAV* lentivirus compared with control and decreased in HuH-7 cells infected with oe-*NRAV* compared with Vector (Fig. 6A). Analysis of ROS by flow cytometry showed a similar trend as the iron accumulation results (Fig. 6B, C). These results suggested that *NRAV* plays a negative regulatory role in ferroptosis in HCC. Furthermore, western blot analysis in Fig. 6D revealed that *SLC7A11* and GPX4 expressions were decreased in hepG2 cells infected with sh-*NRAV* lentivirus compared with control and increased in HuH-7 cells infected with lv-*NRAV* compared with Vector. The opposite trends were observed in ACSL4 expressions, and the most obvious change is *SLC7A11*. The potential downstream miRNA of *NRAV* was found through the annolnc2 database, in which *miR-375-3P* was reported to regulate the key molecule *SLC7A11* of ferroptosis [24]. However, the specific mechanism by which *NRAV* regulates the *miR-375-3P*/*SLC7A11* axis has not been explored. Through bioinformatics methods, we discovered the binding site of *NRAV* and *miR-375-3P* (Fig. 6E). We conducted immunoprecipitation assays using the AGO2 antibody and found that the AGO2 antibody was able to enrich both endogenous *miR-375-3P* and *NRAV* (Fig. 6F). To verify the binding of *NRAV* and *miR-375-3P*, we constructed two *NRAV* luciferase reporter plasmids: wild type and *miR-375-3P* binding site mutant (Fig. 6G). The luciferase activity of the wild type can be significantly inhibited by *miR-375-3P* mimics, but the luciferase activity of the mutant has no significant change (Fig. 6H).

Moreover, the knockout of *NRAV* significantly increased *miR-375-3P* expression, while the overexpression of *NRAV* significantly reduced the expression of *miR-375-3P* (Fig. 6I, J). western blot analysis confirmed that the *miR-375-3P* inhibitor and mimic reversed the alterations in *SLC7A11* caused by alterations in *NRAV*(Fig. 6K). These results prove that *NRAV* promotes iron export and ferroptosis resistance by regulating the *miR-375-3P*/*SLC7A11* axis.

### High expression of *NRAV* is associated with poor prognosis in HCC patients

**Fig. 7:**
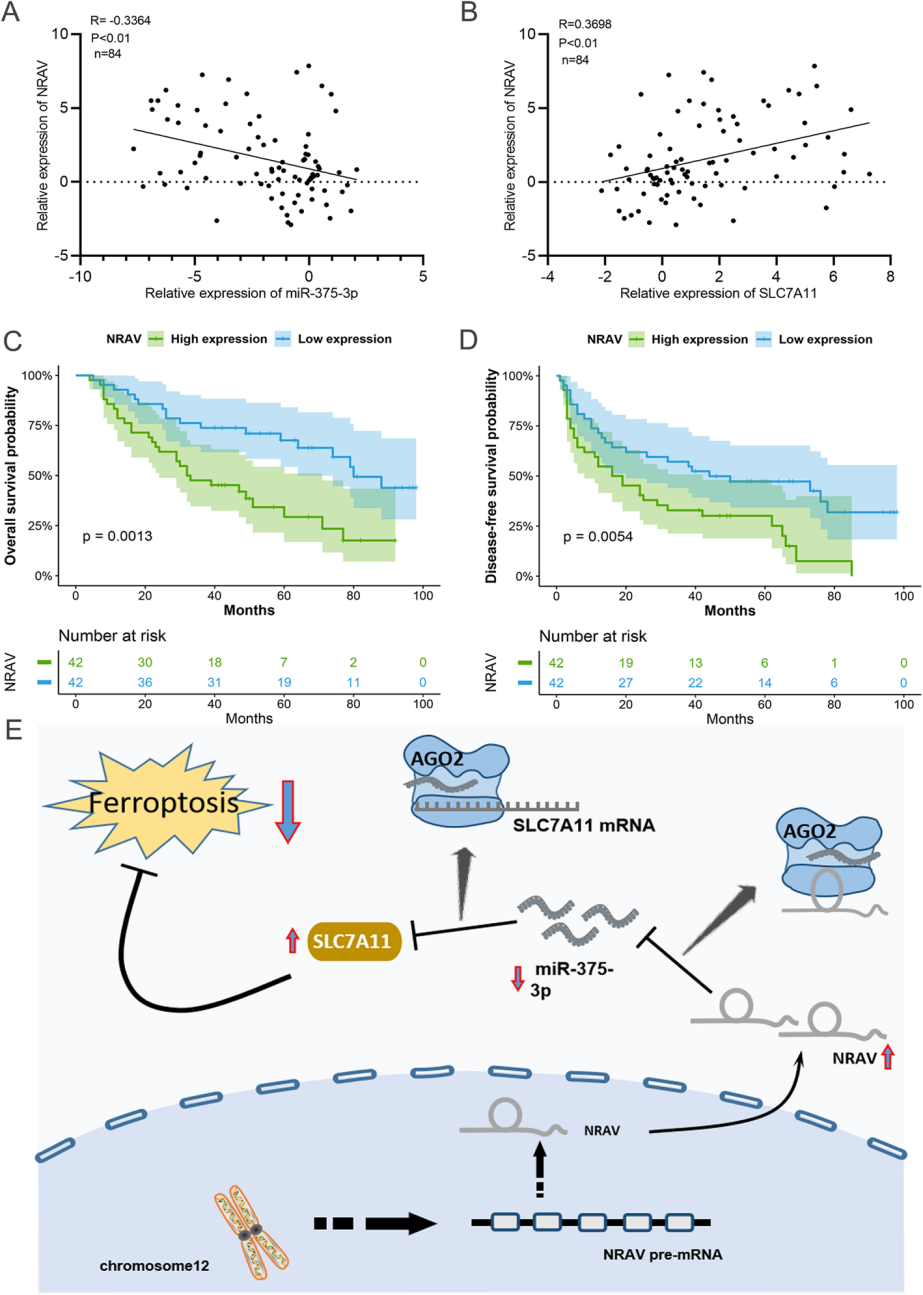
Overexpression of *NRAV* is associated with poor prognosis in HCC patients. **A**-**B** The correlation between *NRAV* and *miR-375-3P* or *SLC7A11* in HCC was analyzed by Pearson correlation analysis. **C**-**D** Overall, disease-free survival analyses were performed to assess the impact of *NRAV* expression high and low in HCC patients. **E** Schematic illustration of the *NRAV*/*miR-375-3P*/*SLC7A11* axis in HCC cells. Results are presented as mean ± SD. *P < 0.05; **P < 0.01, ***P < 0.001, ****P < 0.0001

Through the analysis of q-PCR results of clinical samples, the results showed that the expression of *NRAV* was negatively correlated with *miR-375-3P* and positively correlated with *SLC7A11* (Fig. 7A, B). To further study the clinical significance of *NRAV* in HCC, the clinical characteristics of patients with high *NRAV* expression in our hospital showed that the clinical outcome of HCC patients with high *NRAV* expression was worse than that of patients with low *NRAV* expression (Fig. 7C, D). The pattern of ferroptosis regulated by *NRAV* is shown in Fig. 7E. These results suggest that the high expression of *NRAV* may predict a poor prognosis in patients with HCC.

## Discussion

In recent years, great progress has been made in treating HCC. It is still one of the leading causes of cancer-related deaths worldwide, suggesting an urgent need to develop new treatments for HCC [4]. Ferroptosis is thought to play an important role in immunotherapy. Many kinds of lncRNA are thought to have abnormal expression and participate in cancer progression by binding to genetic material and encoding proteins or other small molecular peptides [3, 25]. Therefore, we developed a lncRNA prognostic risk model combined with ferroptosis. This study identified 84 genes and 52 significant lncRNA associated with ferroptosis. Finally, 6 lncRNA characteristic models related to ferroptosis were established, most of which have been reported to affect the progression of HCC and are associated with poor prognosis, including *NRAV* [26]. He et al. found that *ZFPM2-AS1* acts as a miRNA sponge and promotes cell invasion by regulating *miR-139/GDF10* in HCC [27], Ma et al. Found that *DANCR* was up-regulated in tumor tissues and plasma of patients with HCC, and its expression was highly correlated with microvascular and liver capsule invasion of HCC [28], MKLN1-AS [29], *LNCSRLR* [30] and *AL137186.2* [31] also are associated with the prognosis of patients with HCC, which are consistent with our findings. Moreover, after correcting for traditional clinical risk indicators, the six-ferroptosis-related lncRNAs signature model was shown to be an independent predictive factor for HCC. This result suggested that the six-ferroptosis-related lncRNAs signature could reliably predict the prognosis of HCC patients.

Although adjuvant chemotherapy, immunotherapy, and other anticancer therapies have shown encouraging results in early cancer treatment, the middle and later stages of treatment are challenging in most cases. Therefore, it is imperative to find a more effective cancer treatment strategy. Ferroptosis has long been proven to be closely related to immunotherapy [32, 33], so we further explored the connection between the model and immunity. We studied the difference in immune checkpoint expression between the two groups and found significant differences in T cell function, including checkpoint (inhibition), cytolysis, HLA, inflammatory regulation, co-stimulation, co-inhibition, and type II INF response between low-risk and high-risk groups. Given the importance of immunotherapy based on checkpoint inhibitors, we further discussed the difference in immune checkpoint expression between the two groups. The results showed significant differences in the expression of PDCD-1 (PD-1), CTLA4, HHLA2, and CD44, between the two groups. Most of them play a key role in immunotherapy.

To further study the regulation of lncRNA on ferroptosis, we selected the lncRNA *NRAV* with the most pronounced effect on prognosis for validation. It has been proved that *NRAV* has a clear relationship with the occurrence and development of HCC. Wang et al. have proved that *NRAV* can promote the occurrence and development of HCC through Wnt/ β-catenin signal pathway [26]. In addition, several HCC-related prognostic models have shown that *NRAV* is related to immunity and cell death [34–36]. Our results show that the change of *NRAV* expression can significantly affect the ferroptosis-related proteins *SLC7A11*, GPX4, and ACSL4, especially *SLC7A11*, and can change the Fe^2+^ content and ROS level in HCC cells. Through a series of tests, we found that *NRAV* can change the expression of *SLC7A11* by binding to *miR-375-3P*. Our results show that *NRAV* promotes the occurrence and development of HCC and affects ferroptosis in HCC through the *miR-375-3P*/*SLC7A11* axis.

Compared with other lncRNA prognostic models related to ferroptosis in HCC [37–39], this study has more in-depth basic research, a larger sample size, and stronger prediction accuracy. Through lasso regression analysis and the performance of lncRNA in HCC, we selected the most representative ferroptosis-related lncRNAs-*NRAV* and verified it, and first found the regulation of *NRAV* via *miR-375-3P*/*SLC7A11* axis on ferroptosis, but this study also had some defects, including the lack of research depth, and the mechanism research is relatively simple. At the same time, due to the difficulty of clinical data collection and verification, the number of related clinical samples is relatively small. In the future, we will continue to study the role of *NRAV* in immunity to improve the mechanism of ferroptosis and immunotherapy.

## Conclusion

In conclusion, we developed a new model that can be used for clinical prognosis and demonstrated that *NRAV* could affect ferroptosis in HCC cells through the *miR-375-3P*/*SLC7A11* axis and then affect the occurrence and development of HCC. Our findings expand the mechanism of ferroptosis regulation in HCC and provide new markers for HCC prognosis.

### Supplementary Information

**Supplementary Material 1.**

**Supplementary Material 2.**

**Supplementary Material 3.**

**Supplementary Material 4.**

## Data Availability

All data are available via the corresponding author. The datasets analysed during the current study are available in the TCGA database (https://portal.gdc.cancer.gov/) and ICGC database (https://dcc.icgc.org/).

## Abbreviations

GEO: Gene Expression Omnibus
ICGC: International Cancer Genome Consortium
RIP: RNA immunoprecipitation
RCD: Regulatory cell death
TCGA: The Cancer Genome Atlas
DEGs: Differentially expressed genes
KEGG: Kyoto Encyclopedia of Genetics and Genomes
GO: Genetics Ontology
MSigDB: Molecular Signature Database
ECL: Enhanced chemiluminescence
CDX: Cell line-derived xenograft
OS: Overall survival
DFS: Disease-free survival
CCK-8: Cell Counting Kit-8
GSEA: Gene set enrichment analyses
CPC: Coding-potential computer
