# Supplementary material for: Ferroptosis-related lncRNA NRAV affects the prognosis of hepatocellular carcinoma via the miR-375-3P/SLC7A11 axis
Source: BMC Cancer. 2024 Apr 18;24:496. doi: 10.1186/s12885-024-12265-y (PMC11027313; doi:10.1186/s12885-024-12265-y)
Supplement: Supplementary file 1 — Supplementary Material 1. [file 12885_2024_12265_MOESM1_ESM.pdf]

Table S1. The clinical information of patients in the TCGA dataset.

Variable	Number of samples
**Gender**	
Male/Female	255/122
Age at diagnosis	
≤65/>65/NA	235/141/1
**Grade**	
G1/G2/G3/G4/NA	55/180/124/13/5
**Stage**	
I/II/III/IV/NA	175/87/86/5/24
**T**	
T1/T2/T3/T4/NA	185/95/81/13/3
**M**	
M0/M1/NA	257/4/101
**N**	
N0/N1/N2/N3/NA	172/67/167/8/116
