# Supplementary material for: Ferroptosis-related lncRNA NRAV affects the prognosis of hepatocellular carcinoma via the miR-375-3P/SLC7A11 axis
Source: BMC Cancer. 2024 Apr 18;24:496. doi: 10.1186/s12885-024-12265-y (PMC11027313; doi:10.1186/s12885-024-12265-y)
Supplement: Supplementary file 2 — Supplementary Material 2. [file 12885_2024_12265_MOESM2_ESM.docx]

Table S2. The sequences (5'-3') of primers and shRNA target sequences related to methods.

| NRAV-F(homo） | 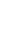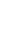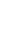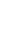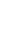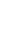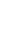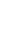ACAATGTGAGGATTAAGTGAGATGA |
| --- | --- |
| NRAV-R(homo） | AAGGTGAATAGTGCCAAGGTT |
| miR-375-F(homo） | AAGCTTTGTTCGTTCGGCTC |
| miR-375-R(homo） | GTATCCAGTGCGAATACCTC |
| SLC7A11-F(homo） | TCTCCAAAGGAGGTTACCTGC |
| SLC7A11-R(homo） | AGACTCCCCTCAGTAAAGTGAC |
| GAPDH-F(homo） | TGTGGGCATCAATGGATTTGG |
| GAPDH-R(homo） | ACACCATGTATTCCGGGTCAAT |
| U6-F(homo） | CTCGCTTCGGCAGCACA |
| U6-R(homo） | AACGCTTCACGAATTTGCGT |
| sh1-NRAV target sequence | GAGGTTCAAAGAGATGAAGTC |
| sh2-NRAV target sequence | GGTCTGACTCTACAGTCCCAC |
| sh3-NRAV target sequence | GGAGCAGTTAGAGGCTATTGC |
| shNC-NRAV target sequence | TTCTCCGAACGTGTCACGT |

| Gene Name | Sequence |  |
| --- | --- | --- |
|  | sense（5'-3'） | antisense（5'-3'） |
| hsa-miR-375-3p-mimics | UUUGUUCGUUCGGCUCGCGUGA | UCACGCGAGCCGAACGAACAAA |
| hsa-miR-375-3p-inhibitors |  | UCACGCGAGCCGAACGAACAAA |
| mimics-NC | UUCUCCGAACGUGUCACGU | GUGACACGUUCGGAGAAUU |
| inhibitor-NC |  | GUGACACGUUCGGAGAAUU |
