# Supplementary material for: Ferroptosis-related lncRNA NRAV affects the prognosis of hepatocellular carcinoma via the miR-375-3P/SLC7A11 axis
Source: BMC Cancer. 2024 Apr 18;24:496. doi: 10.1186/s12885-024-12265-y (PMC11027313; doi:10.1186/s12885-024-12265-y)
Supplement: Supplementary file 3 — Supplementary Material 3. [file 12885_2024_12265_MOESM3_ESM.docx]

**Supplementary Materials**


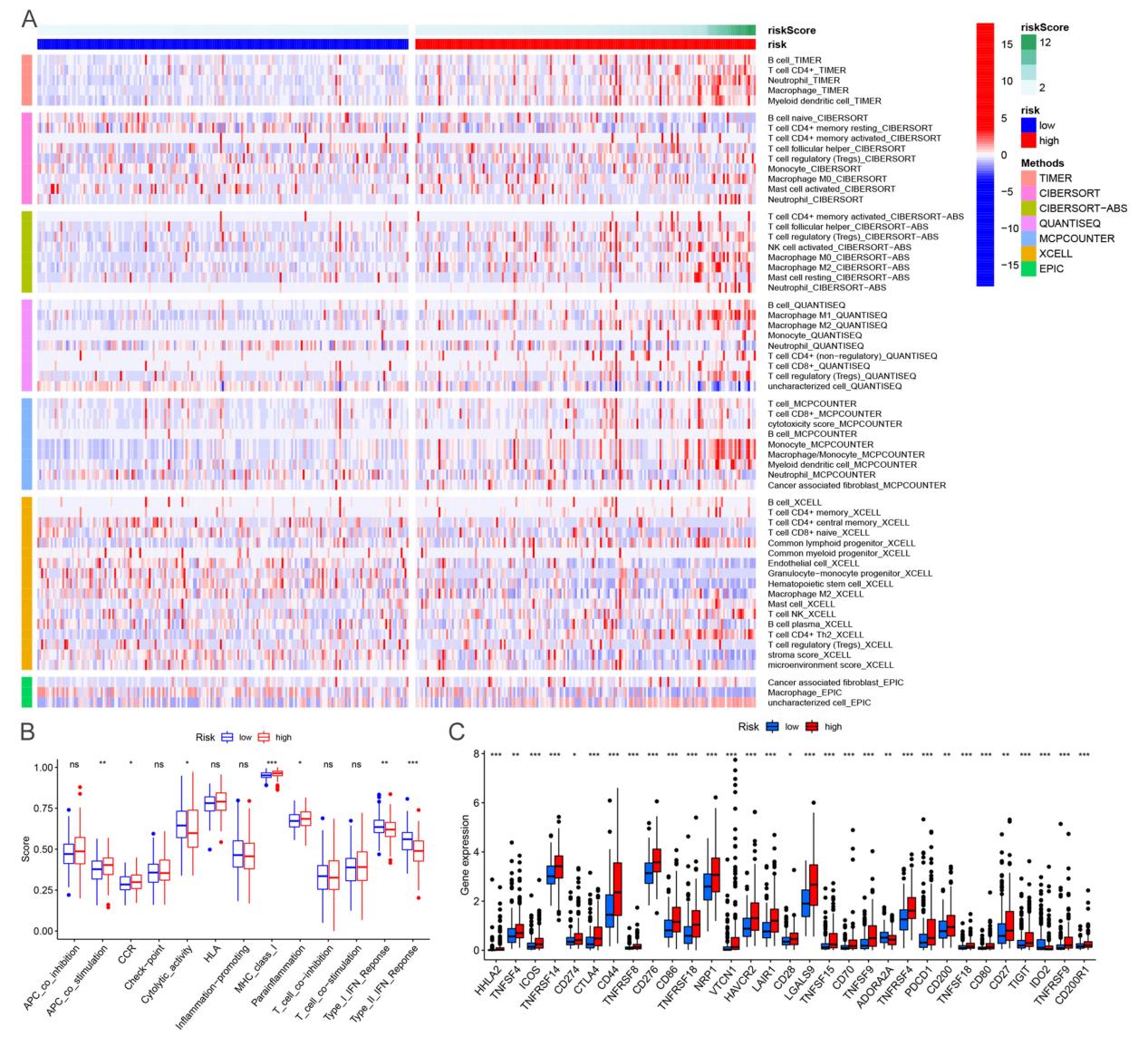


Figure S1. A. Heatmap for immune responses based on CIBERSORT, ESTIMATE, quanTIseq, xCell, MCP-counter, EPIC, and TIMER algorithms among the high and low-risk groups. B. The correlation between immune cell subpopulations and related functions was evaluated using ssGSEA. C. Expression of common immune checkpoints between the high and low-risk HCC patients.


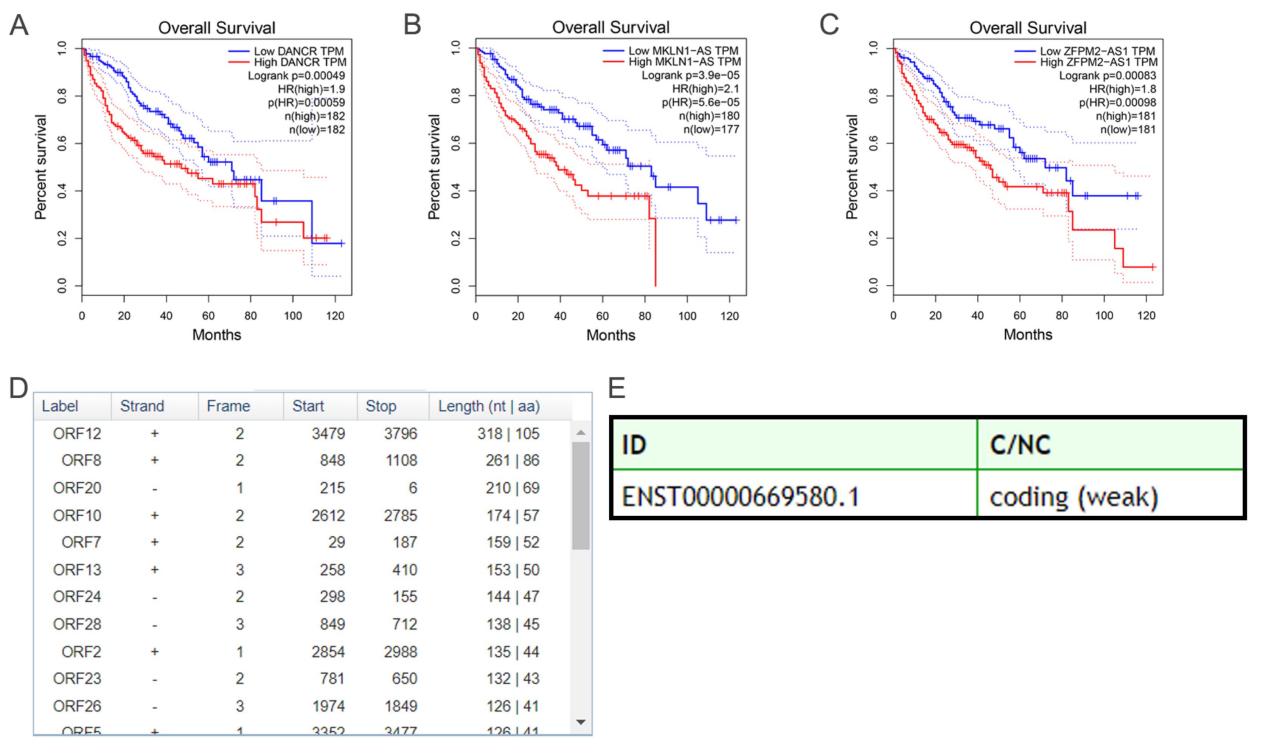


Figure S2. A-C. Overall survival of HCC patients (N=362) from the Gepia project with high or low expression levels. D. The protein-coding potential analysis of *NRAV* was performed using the ORF finder from NCBI. E. Using CPC 2.0 to predict the coding capability of *NRAV*. Table S1. The list of 174 ferroptosis-related DEGs. Table S2. The list of primer sequences. (Supplementary Materials)
