# Supplementary figures and images for: Ferroptosis-related lncRNA NRAV affects the prognosis of hepatocellular carcinoma via the miR-375-3P/SLC7A11 axis
Source: BMC Cancer. 2024 Apr 18;24:496. doi: 10.1186/s12885-024-12265-y (PMC11027313; doi:10.1186/s12885-024-12265-y)

fig4D

NRAV

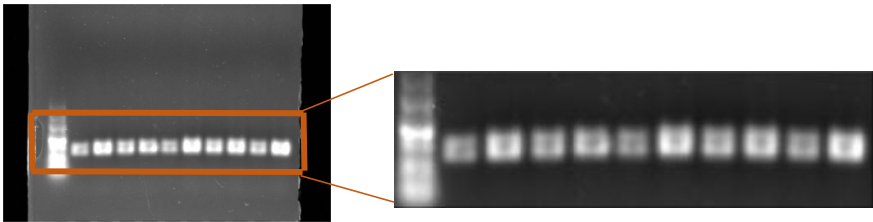

GAPDH

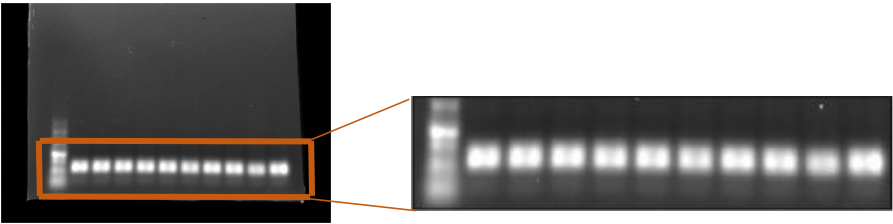

fig4E

NRAV

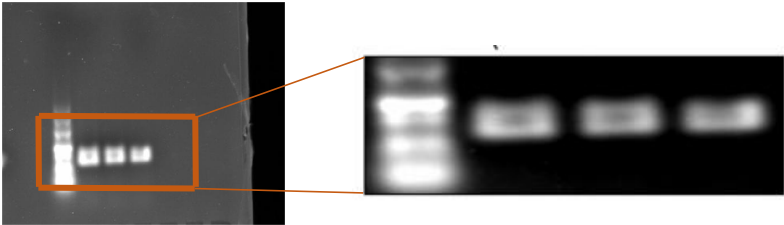

GAPDH

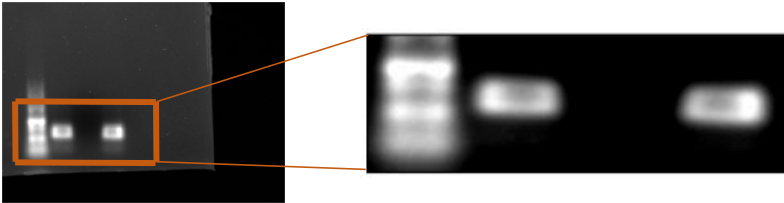

U6

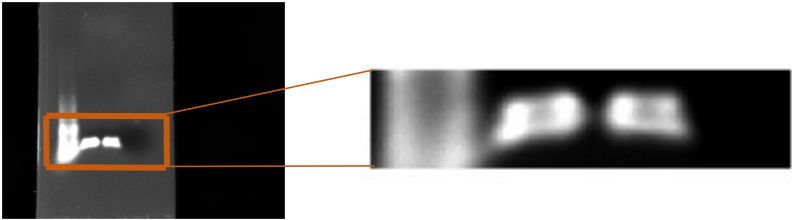

fig4F

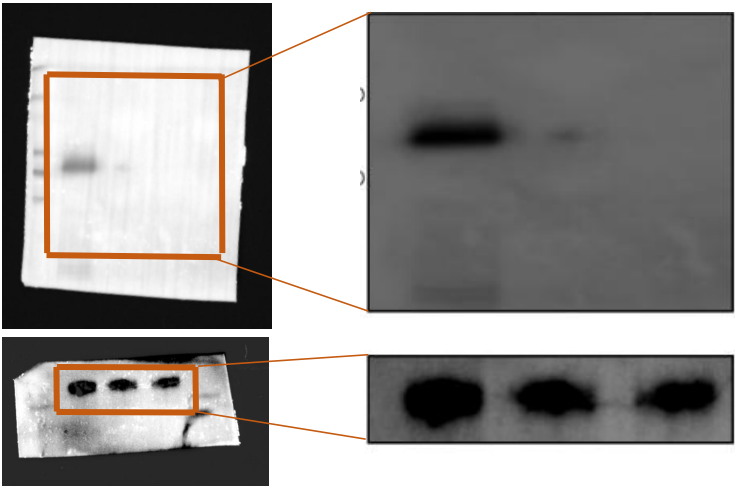

fig6D

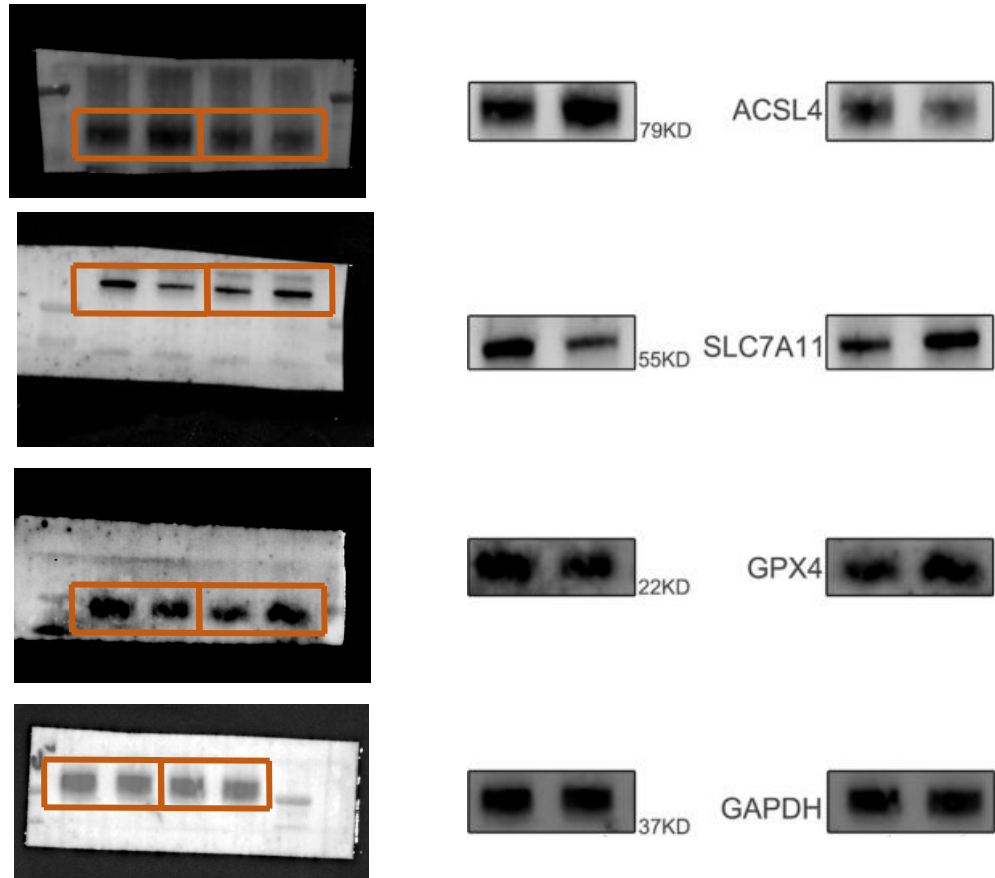

fig6K

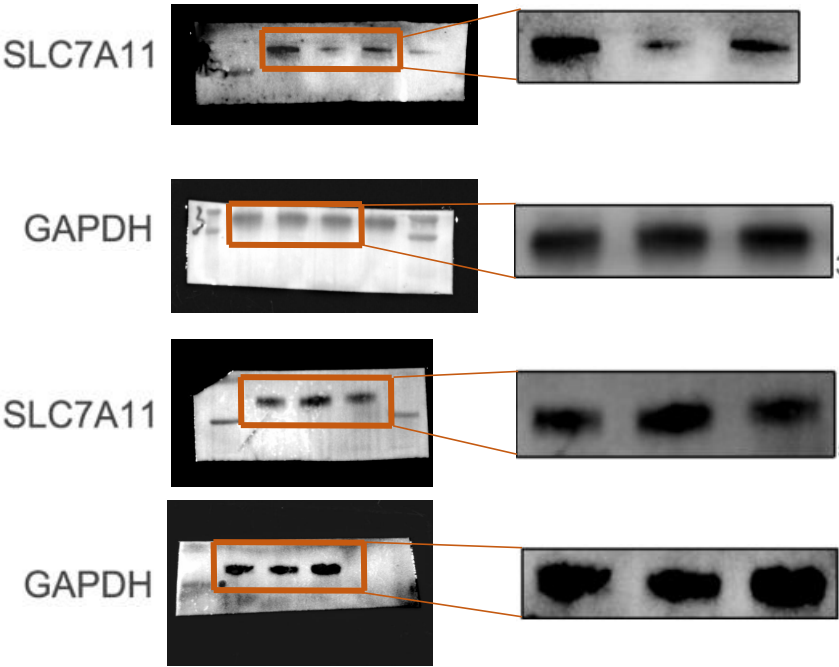

Supplement: Supplementary file 4 — Supplementary Material 4. [file 12885_2024_12265_MOESM4_ESM.pdf]
